# Life cycle and larval feeding strategies of the mangrove firefly *Pteroptyx tener* (Coleoptera, Lampyridae, Luciolinae): implications for conservation of a vulnerable species

**DOI:** 10.7717/peerj.21575

**Published:** 2026-08-05

**Authors:** Parichart Laksanawimol, Rinrada Jundasri, Anchana Thancharoen

**Affiliations:** 1 Faculty of Science, Chandrakasem Rajabhat University, Bangkok, Thailand; 2 IUCN Firefly Specialist Group, Gland, Switzerland; 3 Research and Lifelong Learning Center for Urban and Environmental Entomology, Institute for Advanced Studies, Kasetsart University, Bangkok, Thailand; 4 Department of Entomology, Kasetsart University, Bangkok, Thailand

**Keywords:** IUCN red list, Rearing, Feeding behavior, Mangrove, Vulnerable species, Gregarious feeding behavior, Thailand, Larval instars, Biology

## Abstract

*Pteroptyx tener* is a mangrove-associated firefly known for its synchronous flashing behavior and is currently classified as Vulnerable on the International Union for Conservation of Nature (IUCN) Red List. Despite its ecological and economic importance, its larval biology remains poorly understood. This study aimed to describe the life cycle and larval feeding ecology of *Pteroptyx tener* under controlled conditions. Adults were collected from a mangrove river system in southern Thailand and reared in two experimental trials under laboratory conditions. Eggs, larvae, and pupae were maintained on moist cotton substrates, and larvae were fed juvenile golden apple snails (*Pomacea canaliculata*) throughout development. Larval feeding behavior, developmental duration, survival rates, and morphological characteristics were recorded across all instars. The complete life cycle lasted approximately 7–8 months and comprised egg, larval, pupal, and adult stages, with no significant difference between sexes. Survival was high during early larval stages but declined markedly in later instars and during the prepupal transition, likely due to unsuitable pupation substrates. First-instar larvae exhibited gregarious feeding behavior, while larvae showed temporary starvation tolerance. These findings indicate multiple adaptive traits, including gregarious feeding, developmental plasticity, and trophic flexibility. These results provide a basis for conservation implementation, emphasizing habitat-based management strategies that integrate mangrove restoration, prey resource conservation, and control of anthropogenic stressors to support population persistence of *Pteroptyx tener*.

## Introduction

Fireflies (Coleoptera: Lampyridae) are a charismatic group of bioluminescent beetles that produce light for sexual communication and predator defense, and are widely distributed across tropical and temperate regions worldwide. The genus *Pteroptyx* Olivier is distributed across the Old World, including Southeast Asia, New Guinea, Australia, and Hong Kong (Ballantyne et al., 2011; Ballantyne & McLean, 1970; Jusoh et al., 2018; Sriboonlert et al., 2015). Several species are well known for their synchronous flashing displays, in which aggregations of adults emit courtship signals on trees along estuarine riverbanks (Jaikla et al., 2020; Jusoh, Hashim & Ibrahim, 2010a; Wong, 2022). However, flashing behavior varies among species, and not all *Pteroptyx* exhibit strict synchrony; for example, *Pteroptyx valida* displays asynchronous flashing (Jusoh et al., 2018; Prasertkul, 2018). When large numbers of individuals congregate and communicate through rhythmic flashes on mangrove trees, they produce conspicuous displays that have become well-known attractions in many regions (Lewis et al., 2021; Prasertkul, 2018; Thancharoen, 2012). As a result, habitats supporting *Pteroptyx* fireflies contribute to a multi-million-dollar ecotourism industry in countries such as Malaysia and Thailand, providing sustainable income for local communities (Lewis et al., 2021; Thancharoen, 2012). Because of their strong habitat specialization and close association with biodiverse mangrove habitats, *Pteroptyx* fireflies are often considered indicators of mangrove ecosystem health and may also function as umbrella species for mangrove conservation (Boonloi et al., 2025).

However, *Pteroptyx* populations are increasingly threatened by anthropogenic pressures, particularly the conversion of mangrove forests into oil palm plantations, aquaculture ponds, and urban development (Idris et al., 2021; Jaikla et al., 2020; Lewis et al., 2021, 2020). Artificial light associated with urbanization and tourism infrastructure can disrupt nocturnal courtship signaling (Lewis et al., 2024; Thancharoen & Masoh, 2019). In addition, pesticide runoff and water pollution—particularly elevated ammonia-nitrogen concentrations—may affect firefly survival (Cheng et al., 2021; Faudzi et al., 2021; Leong, Tan & Mustafa, 2007; Lewis et al., 2020). Due to these pressures, several *Pteroptyx* species are currently classified as threatened on the IUCN Red List. Four species from Southeast Asia—*Pteroptyx bearni*, *Pteroptyx malaccae*, *Pteroptyx tener*, and *Pteroptyx valida*—are categorized as Vulnerable (Jusoh et al., 2024; Nada et al., 2024a, 2024b; Thancharoen et al., 2024).

*Pteroptyx tener* is the most common *Pteroptyx* species inhabiting low-salinity riverine mangrove ecosystems in Malaysia (Wong, 2022). Its synchronous flashing displays occur mainly on *Sonneratia caseolaris* (Asri et al., 2021; Jusoh et al., 2018), although observations on other display tree species have also been reported (Azmi, Zainudin & Salam, 2015; Jusoh et al., 2018; Poukin, Mahadimenakbar & Mohamed, 2023). In contrast to its relatively widespread occurrence in Malaysia, *Pteroptyx tener* appears to be rare in Thailand, where it has previously been recorded from only a single locality along the Tapi River in Surat Thani Province (Sriboonlert et al., 2015). In the present study, we report a new locality for *Pteroptyx tener* from the Trang River, providing an additional distribution record for this species in southern Thailand.

Although considerable research has been conducted on *Pteroptyx tener* in various aspects, including taxonomy (Boonloi et al., 2025; Jusoh et al., 2018), flashing behavior (Case, 1980; Ohba & Wong, 2004), geographical distribution (Abu Seri & Abd Rahman, 2022; Dawood & Saikim, 2016; Jaikla et al., 2020; Sriboonlert et al., 2015), habitat characteristics and abundance (Abdullah et al., 2021; Abu Seri & Abd Rahman, 2024, 2025; Jusoh, Hashim & Ibrahim, 2010a, 2010b; Nada et al., 2023), population monitoring (Khoo et al., 2009; Kirton et al., 2012), feeding ecology (Cheng et al., 2017) and ecotourism and conservation (Lewis et al., 2021; Nallakumar, 2003), information on its larval biology remains limited as is generally the case in lampyrid beetles, due to the challenges of successfully rearing larvae to the adult stage (Riley, Rosa & Lima da Silveira, 2021).

According to Riley, Rosa & Lima da Silveira (2021), lampyrid larvae are predominantly predatory, using specialized grooved mandibles to inject neurotoxic venom that immobilizes prey, followed by extra-oral digestion. Most species feed on soft-bodied invertebrates, including gastropods, termites, and earthworms, although prey preferences vary among species and habitats. While many firefly larvae are considered generalist predators, some terrestrial species exhibit a greater degree of specialization, particularly on earthworms. However, observations of larval feeding behavior in natural habitats remain limited, and much of the current knowledge is derived from laboratory studies. Predatory behavior has therefore been investigated primarily under laboratory conditions, where congregative feeding on a single prey item has been documented in the aquatic species, *Aquatica leii* (Fu & Meyer-Rochow, 2012). Despite these observations, comprehensive information on larval feeding strategies remains limited, particularly for the genus *Pteroptyx*.

Preliminary laboratory observations on the breeding of *Pteroptyx tener* were reported by Nada & Kirton (2004), but detailed information on its biology remains limited. This study therefore describes the life cycle and larval feeding behavior of *Pteroptyx tener* under controlled conditions. These findings provide new ecological insights into the biology of this mangrove firefly and contribute to the conservation and management of its natural populations.

## Materials and Methods

### Firefly collection

A new locality record of *Pteroptyx tener* on the Andaman coast of Thailand was identified by Mr. Saichon Sohsiw and is documented here for the first time. The population was discovered along the riparian zone of the Trang River in Kantang District, Trang Province. During the survey period, *Pteroptyx tener* was the only congregating mangrove firefly species observed at the study site. The habitat comprises an extensive nipa palm (*Nypa fruticans*) forest interspersed with scattered *Sonneratia caseolaris* trees, which function as firefly display trees along the riverbank.

Adult of *Pteroptyx tener* (species identification of both males and females confirmed following Jusoh et al. (2018)) were gently collected from display trees using a sweep net and placed in a transparent plastic container (65 mm in height × 95 mm in diameter) with mesh-covered aeration holes and a piece of cotton soaked in honey solution to provide moisture and food. Rearing experiments were conducted in two trials using specimens collected in May and November 2023. The research was approved for animal care and use for scientific research at Kasetsart University (ACKU67-AGR-022), and the corresponding author obtained an animal use license (U1022522558).

### Rearing the firefly

The fireflies were reared at the Department of Entomology, Faculty of Agriculture, Kasetsart University (Bangkok, Thailand) under an ambient temperature of 25–27 °C and a 12:12 h light:dark photoperiod. Two experimental trials were conducted from May to December 2023 (trial 1) and from November 2023 to April 2024 (trial 2).

Females were separated into oviposition containers and provided with a 30% honey solution on a moist cotton pad as a food source, along with a separate cotton pad maintained at 70–80% moisture as an oviposition substrate. After oviposition, the substrate was replaced daily to obtain synchronously aged eggs. The minute and fragile eggs were handled gently using a small brush. Upon hatching, larvae were maintained under two substrate conditions. In Trial 1, a Scott 3M sponge cloth was placed at the base of the container and covered with a layer of moist cotton pads, whereas in Trial 2, only a layer of moist cotton pads was used. Substrate moisture was not quantified. Larvae at all stages were fed juvenile golden apple snails (*Pomacea canaliculata*). Egg masses of the snails were collected from ponds and canals at Kasetsart University and incubated until they turned grey, after which the newly hatched juveniles were cleaned under running water using sieving equipment to remove shell debris before being used immediately as prey (see Supplemental File for snail preparation procedures, Fig. S1). Larvae tended to remain within folded areas of the cotton pads. The pads were moistened daily and replaced every 2–3 days. Maintenance was carried out throughout all larval stages until pupation, except during pre-ecdysis curling behavior, when larvae were not disturbed. Pupation occurred on the moist cotton substrate.

### Larval feeding behavior

Individual rearing of L1 was largely unsuccessful, with low survival observed under solitary conditions. Therefore, L1 were reared in groups, where they exhibited aggregative feeding behavior. Juvenile golden apple snails were provided at approximately 10–30 individuals per rearing box to ensure sufficient food availability. Seven clutches of the same age were used to examine density effects. High-density groups consisted of 102, 105, 111, and 122 larvae per clutch, whereas low-density groups contained 22, 43, and 45 larvae per clutch. Each clutch of newly hatched larvae was observed daily to record feeding behavior until development to the second instar. Feeding events were categorized into four behavioral types: solitary feeding (one larva per prey), small-group feeding (2–5 larvae), large-group feeding (6–9 larvae), and mass aggregation feeding (≥10 larvae). The frequency of each feeding type was daily recorded.

After the first molt, L2 were gently transferred individually to new rearing containers to monitor feeding activity and development through to the sixth instar. Six juvenile golden apple snails were provided per larva and replaced daily. Empty snail shells, identified as consumed prey, were removed and counted. When pre-ecdysis curling behavior was observed, the cleaning and feeding were temporarily stopped until molting occurred. The duration of the non-feeding period prior to curling behavior was recorded as the pre-molt feeding cessation period.

### Developmental time and measurements

Each developmental stage in the life cycle of *Pteroptyx tener* was examined from oviposition to adult emergence. Larval stage duration was calculated from the day after each molt to the day prior to the subsequent molt, including the pre-ecdysis curling behavior. Sex-specific duration was investigated for the larval, pupal, and adult stages. Larval duration by sex was determined by tracking individuals through development to adult, when sex could be identified. Survival rate (%) from egg to adult was calculated as the proportion of individuals surviving to each stage relative to the initial number of individuals.

All larval measurements were taken for L1–L6 using a Leica EZ4 W stereo microscope (Leica, Germany). Body length, body width, protergum length, and protergum width were measured following Boonloi et al. (2025), in which specimens were chilled on ice for 5–10 min to minimize movement before measurement.

### Data analysis

All biological parameters, including morphometric measurements, duration of each developmental stage, feeding events, and number of consumed snails, were analyzed and presented as mean ± SD. Independent-samples t-tests (*p* < 0.05) were performed to compare the mean developmental duration between males and females. All statistical analyses were conducted using SPSS version 14 (SPSS for Windows, Chicago, IL, USA: SPSS Inc.).

## Results

### Basic biology and life cycle

**Figure 1:**
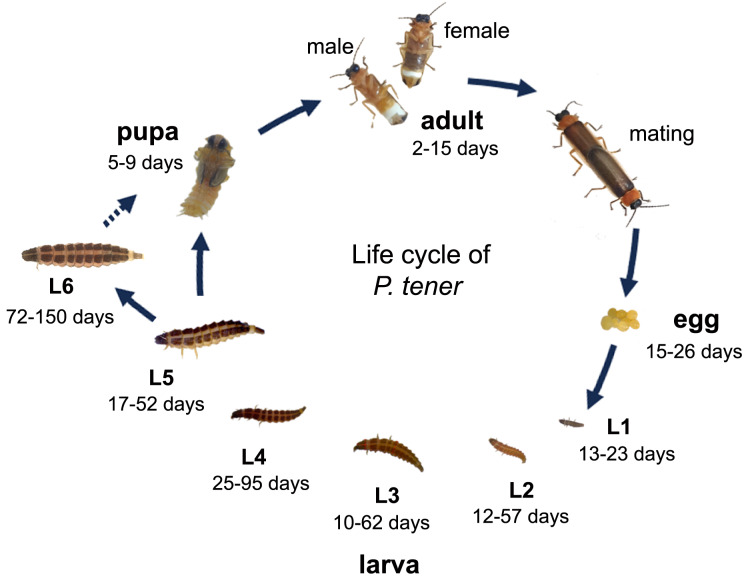
Life cycle of *Pteroptyx tener*. L indicates larval instars.

**Figure 2:**
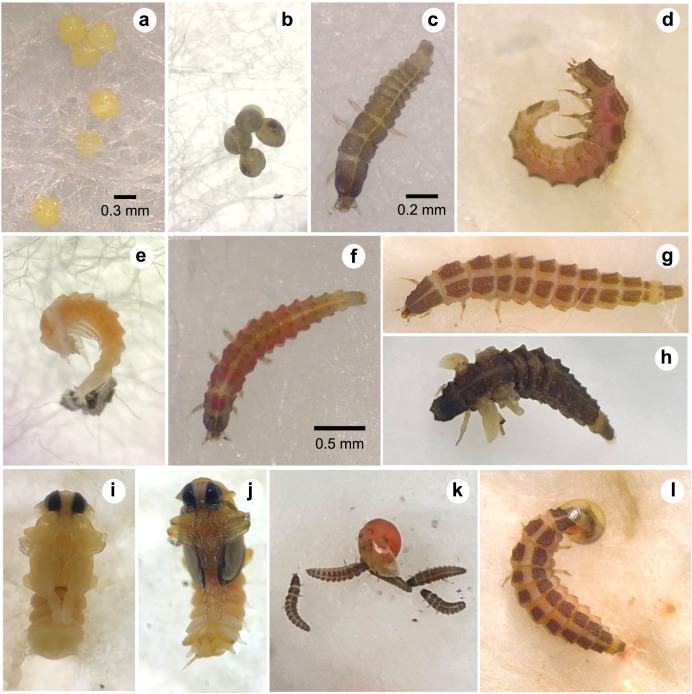
Characteristics and behavior of *Pteroptyx tener*: (A) eggs; (B) nearly hatched eggs; (C) first-instar larva (L1); (D) larva in C-shaped posture; (E) molting larva; (F) newly molted larva; (G) fourth-instar larva (L4); (H) abnormal larva; (I) pupa on day 4; (J) pupa on day 8; (K) aggregative feeding behavior; and (L) solitary feeding behavior.

The life cycle of *Pteroptyx tener* was approximately 7.8 months in males (235.8 ± 48.5 days; range: 151–325 days) and 8.0 months in females (244.2 ± 53.2 days; range: 172–330 days), comprising four distinct developmental stages: egg, larva, pupa, and adult (Figs. 1, 2; Table 1). Although females exhibited a slightly longer mean duration, no significant difference was detected between sexes (t-test: *t* = −0.413, *df* = 23, *p* > 0.05). The two trials exhibited broadly similar patterns in overall life cycle duration; however, notable variation was observed in specific larval instars, particularly L2 and L3. Survival rates from egg to L5 were 65%, 97%, 88%, 76%, 33%, and 25%, respectively, while 100% survival was observed during the pupal and adult stages. The highest mortality occurred during the transition to the pupal stage. Additionally, nematode infection was observed during the L4–L5 stages, resulting in further mortality. The eggs (Fig. 2A) were spherical, pale yellow, and minute, with a mean diameter of 0.38 ± 0.03 mm. The egg surface was smooth, and eggs were laid either singly or in clusters of 2–10. Because the original female specimens were collected from the field and their age and mating status were unknown. Therefore, the number of eggs obtained in this study should be considered an estimate of oviposition under laboratory conditions rather than the total lifetime fecundity. A total of 561 eggs was obtained from nine females, with a maximum of 62.3 eggs per female. As hatching approached, the distinct body pattern of the first-instar larva became visible beneath the chorion (Fig. 2B). The duration of the egg stage averaged 19.7 ± 2.5 days, range 15–26 days. Abnormal eggs were occasionally observed; these were irregular in shape, smaller in size, and orange in coloration, and none hatched successfully.

**Table 1:** Stage-specific developmental duration of *Pteroptyx tener* in males and females.

Stage	Trial 1 (days)		Trial 2 (days)		Combined trials	
	Mean ± SD (N)	Range	Mean ± SD (N)	Range	Mean ± SD (N)	Range
**Duration by developmental stage**						
Egg	20.5 ± 2.7 (214)	16–26	18.9 ± 2.0 (214)	15–22	19.7 ± 2.5 (428)	15–26
L1	18.4 ± 2.5 (44)	13–23	17.6 ± 1.5 (30)	16–21	18.1 ± 2.2 (74)	13–23
L2	23.1 ± 4.4 (41)	12–34	34.1 ± 9.8 (30)	18–57	27.7 ± 8.9 (71)	12–57
L3	23.2 ± 8.9 (42)	10–60	38.5 ± 13.9 (27)	17–62	28.4 ± 13.1 (79)	10–62
L4	60.6 ± 19.7 (42)	31–95	58.5 ± 24.0 (12)	25–91	60.1 ± 20.5 (54)	25–95
L5	33.4 ± 10.4 (15)	17–52	25.8 ± 6.7 (5)	18–34	31.5 ± 10.0 (20)	17–52
L6	N/A	N/A	82.2 ± 21.9 (5)	72–150	82.2 ± 21.9 (5)	72–150
**Duration by sex**						
**L1–L5**						
Male	228.4 ± 38.6 (5)	194–293	172.4 ± 27.5 (7)	115–199	202.9 ± 47.9	115–293
Female	290.0 ± 2.8 (2)	288–292	202.2 ± 48.8 (11)	134–272	206.7 ± 52.6	134–292
Both sexes	246.0 ± 43.6 (7)	194–293	190.6 ± 43.5 (18)	115–272	206.1 ± 49.6 (25)	115–293
**Pupa**						
Male	7.2 ± 1.3 (5)	6–9	6.6 ± 0.8 (7)	6–7	6.8 ± 1.0 (12)	6–9
Female	6.5 ± 0.7 (2)	6–7	7.5 ± 1.2 (11)	5–9	7.3 ± 1.2 (13)	5–9
Both sexes	7.0 ± 1.2 (7)	6–9	7.1 ± 1.1 (18)	5–9	7.1 ± 1.1 (25)	5–9
**Adult**						
Male	7.6 ± 4.0 (5)	3–14	3.9 ± 2.1 (7)	2–7	5.4 ± 3.5 (12)	2–14
Female	10.0 ± 1.1 (2)	9–11	9.1 ± 2.9 (11)	4–15	9.2 ± 2.7 (13)	4–15
Both sexes	8.3 ± 3.6 (7)	3–14	7.1 ± 3.7 (18)	2–15	7.4 ± 3.6 (25)	2–15

**Note:**

Data are shown as the mean ± SD. N/A, Not available.

The larvae were fusiform, slightly dorsoventrally flattened, and membranous with a relatively soft body. The dorsal tergites were darkly pigmented and bore tubercles along their lateral margins. The duration of the larval stage averaged 206.1 ± 49.6 days, range 115–293 days. The larval stage comprised five to six instars, with a markedly smaller L1 (Fig. 2C). Prior to molting, larvae at all instars exhibited pre-ecdysis curling behavior for 3–5 days. During this period, they curled into a C-shaped posture (Fig. 2D), ceased feeding, and remained immobile. Immediately after ecdysis, body coloration was pinkish and gradually darkened to deep brown as the cuticle hardened (Figs. 2E, 2F). The final curling posture, corresponding to the prepupal period, lasted 4.1 ± 0.9 days (range: 2–6 days, *N* = 31). When the L5 served as the final larval stage, L4 (Fig. 2G) had the longest duration among all developmental stages. Most adults developed through five instars (*N* = 20), whereas a few individuals underwent a sixth instar (*N* = 5) with a prolonged developmental period. In this study, none of the sixth-instar larvae successfully developed into pupae. A few final-instar larvae exhibited abnormal thoracic characteristics, characterized by two pairs of wing-like structures (Fig. 2H). The total larval period across all instars averaged 222.8 ± 47.7 days (range: 158–293 days, *N* = 11).

The pupa was exarate and lasted 5–9 days, with a yellowish body coloration. The eyes turned black on day 4 (Fig. 2I), and the wings darkened on days 7–8 (Fig. 2J). The duration of the pupal stage averaged 7.1 ± 1.1 days, range 5–9 days. Although the pupal stage exhibited a high survival rate, abnormal adult emergence was frequently observed, likely due to fine fibers from cotton substrate adhering to the pupal body, which resulted in abnormal adult emergence.

Adults possess an elongate, soft-bodied form, characterized by dark brown elytra and an orange pronotum. Male and female adults exhibited similar body sizes (5.00 ± 0.12 mm, *N* = 12 and 5.03 ± 0.48 mm, *N* = 7, respectively). Adults remained immobile in the pupation area for 1–2 days after emergence. Females laid eggs 2–3 days after mating.

### Morphometric changes across instars

**Figure 3:**
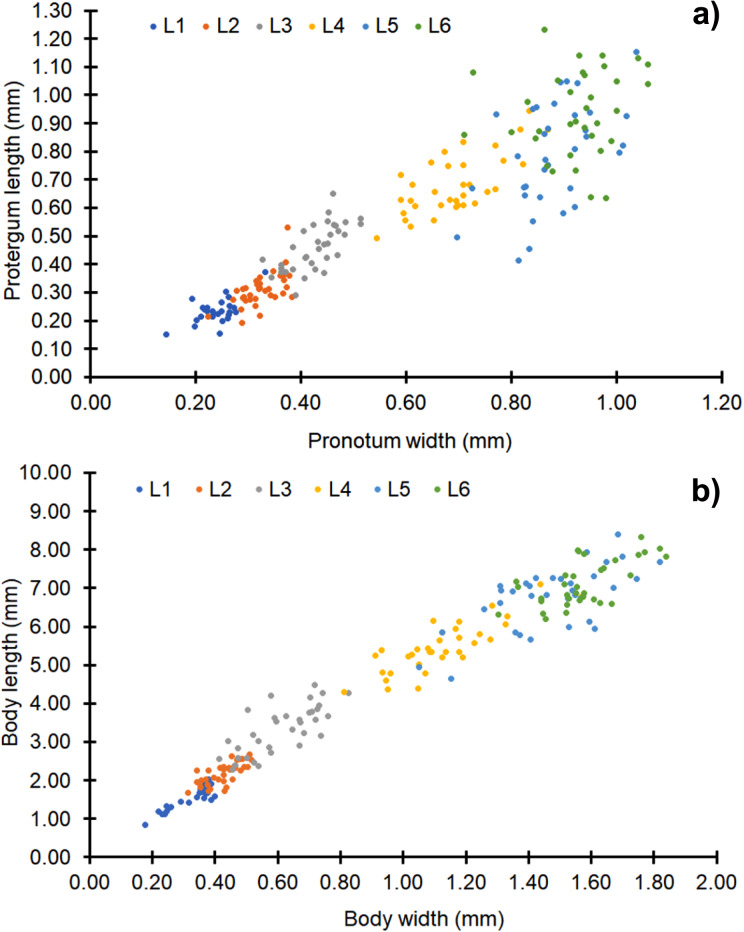
Scatter plots of protergum (A) and body (B) measurements across larval instars (L1–L6) of *Pteroptyx tener*.

Measurements of larval morphology conducted across all developmental instars exhibited distinct differences in protergum and body size were observed among instars (Table 2). The width and length of the protergum, as well as overall body width and length, increased progressively from L1 through L5 (Fig. 3) (One way analysis of variance (ANOVA): protergum, *F* = 179.929, *df* = 5, *p* = 0.000; One way ANOVA: body width, *F* = 600.273, *df* = 5, *p* = 0.000). L1 larvae were minute (0.85–2.03 mm) and difficult to observe and handle with the naked eye. Body size increased progressively through subsequent instars, with L2–L6 measuring approximately 1.4-, 2.2-, 3.6-, 4.4-, and 4.6-fold larger than L1, respectively.

**Table 2:** Protergum and body size of *Pteroptyx tener* across larval stages. Data are presented as mean ± SD (N), with ranges shown in parentheses below.

Stage	Protergum size (mm)		Body size (mm)	
	Width	Length	Width	Length
**L1**	0.24 ± 0.04 (26)	0.23 ± 0.04 (26)	0.32 ± 0.07 (26)	1.51 ± 0.30 (26)
	(0.14–0.33)	(0.15–0.37)	(0.18–0.40)	(0.85–2.03)
**L2**	0.32 ± 0.04 (35)	0.31 ± 0.06 (35)	0.42 ± 0.05 (35)	2.16 ± 0.29 (34)
	(0.22–0.38)	(0.19–0.53)	(0.31–0.52)	(1.67–2.68)
**L3**	0.42 ± 0.05 (35)	0.45 ± 0.08 (35)	0.61 ± 0.11 (35)	3.30 ± 0.63 (34)
	(0.33–0.51)	(0.29–0.65)	(0.41–0.83)	(2.28–4.49)
**L4**	0.69 ± 0.08 (34)	0.68 ± 0.08 (34)	1.11 ± 0.16 (34)	5.44 ± 0.66 (34)
	(0.54–0.87)	(0.49–0.94)	(0.81–1.57)	(4.30–7.10)
**L5**	0.87 ± 0.08 (34)	0.78 ± 0.18 (34)	1.46 ± 0.18 (34)	6.70 ± 0.82 (34)
	(0.70–1.04)	(0.41–1.15)	(1.05–4.64)	(4.64–8.41)
**L6**	0.92 ± 0.08 (34)	0.93 ± 0.15 (34)	1.57 ± 0.13 (34)	7.11 ± 0.59 (34)
	(0.71–1.06)	(0.64–1.23)	(1.31–1.84)	(6.19–8.34)

### Larval feeding behavior

**Figure 4:**
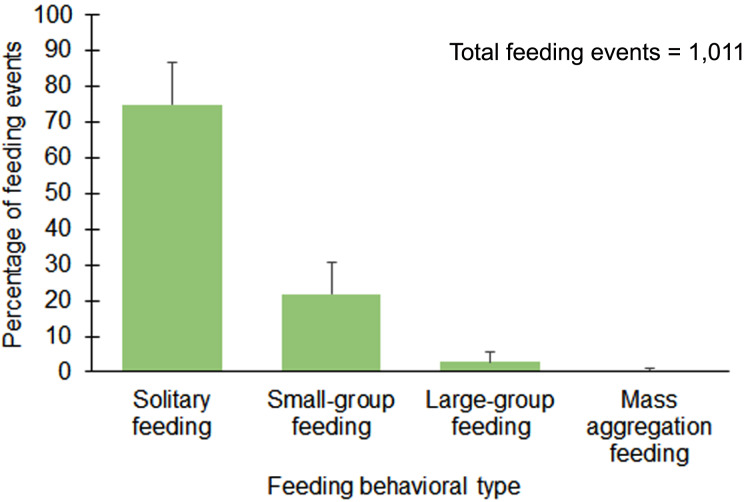
Aggregative feeding behavior of first-instar larvae (L1) of *Pteroptyx tener*, categorized into solitary, small-group, large-group, and mass aggregation feeding.

**Figure 5:**
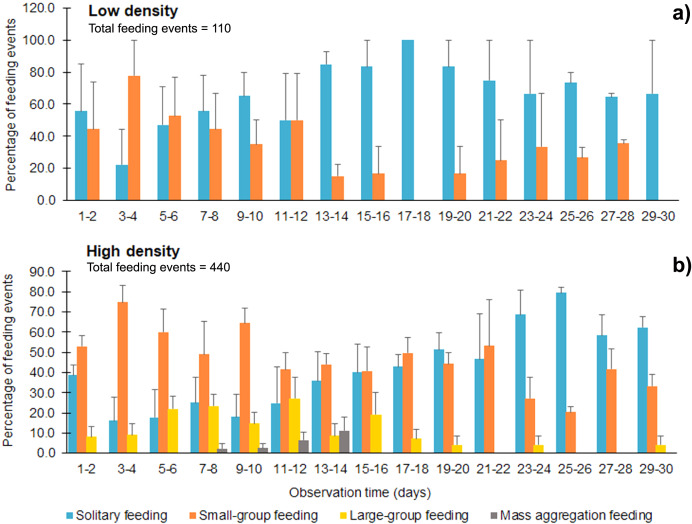
Daily aggregative feeding behavior of first-instar larvae (L1) of *Pteroptyx tener*, categorized into solitary, small-group, large-group, and mass aggregation feeding under low- (A) and high-density (B) conditions.

L1 exhibited aggregative feeding behavior (Fig. 2K), which appeared to enhance survival during this fragile stage. In contrast, individual rearing of L1 resulted in a high mortality rate within 3 days. From 1,011 feeding events, as shown in Fig. 4, most L1 displayed solitary feeding (75%), followed by small-group (22%) and large-group feeding (3%); however, grouping patterns were influenced by larval density (Fig. 5). Under low-density conditions, small-group feeding occurred primarily in larvae less than 10 days old, whereas solitary feeding (Fig. 2L) predominated in older larvae. Large-group and mass aggregations (up to 12 larvae per snail) were observed only under high-density conditions; however, small-group feeding remained the predominant aggregation type.

**Figure 6:**
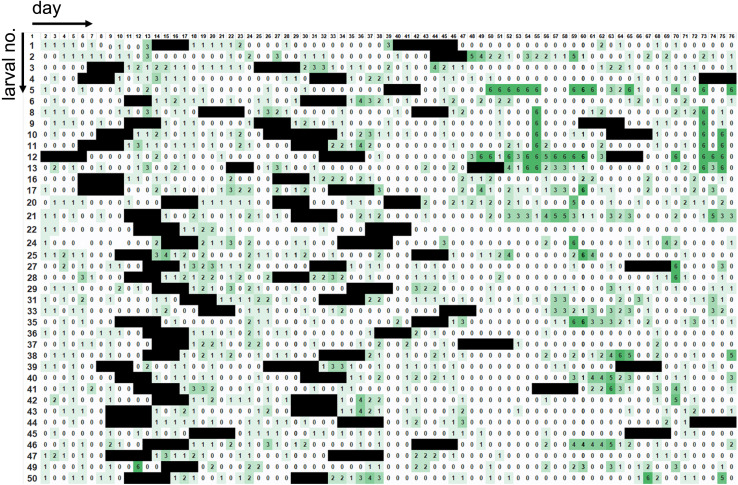
Daily feeding activity of 50 first-instar larvae (L1) over 76 days. Light green indicates a low number of snails consumed, whereas darker shades indicate higher numbers of snails consumed. Dark bars represent periods of pre-molt feeding cessation.

**Figure 7:**
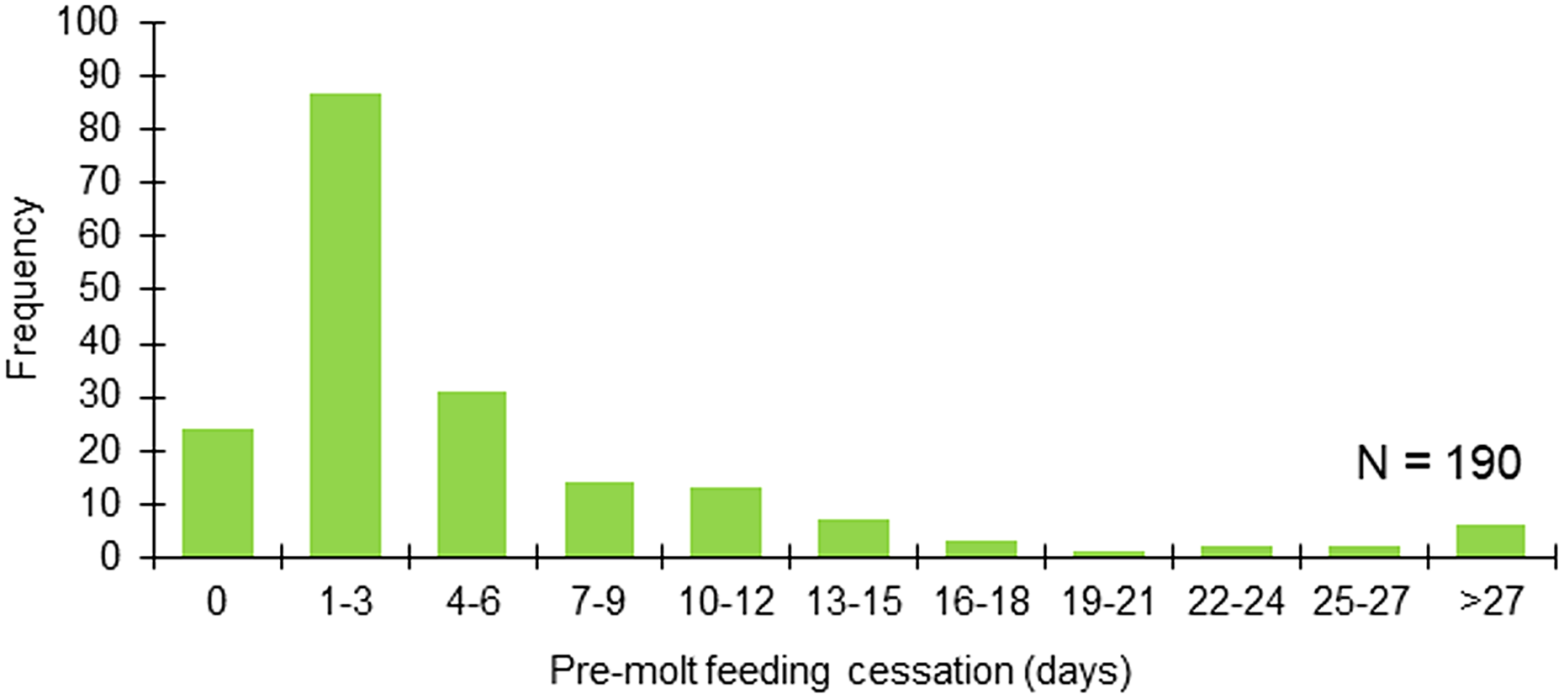
Duration of pre-molt feeding cessation in *Pteroptyx tener* larvae.

During larval development (Fig. 6), individuals did not feed daily and were able to survive without food for more than 20 days, particularly during the pre-molt feeding cessation period, which typically lasted 1–3 days but occasionally extended up to 1 month (Fig. 7). Food consumption increased with larval stage, averaging 12.4 ± 4.4, 13.9 ± 6.8, 26.7 ± 9.7, 31.5 ± 10.0, and 75.6 ± 10.7 snails for L2–L5, respectively (Table 3), totaling approximately 76 snails per individual.

**Table 3:** Stage-specific number of snails consumed by *Pteroptyx tener* larvae, presented as mean ± SD (N), with ranges shown in parentheses below.

	L2	L3	L4	L5	L2-L5
**Trial 1**	10.2 ± 3.0 (32)	10.1 ± 4.2 (40)	25.9 ± 10.0 (30)	33.4 ± 10.4 (15)	77.4 ± 10.1 (23)
	(5–18)	(4–23)	(12–44)	(17–52)	(59–98)
**Trial 2**	15.0 ± 4.3 (27)	19.3 ± 6.3 (27)	28.0 ± 9.4 (17)	25.8 ± 6.7 (5)	73.7 ± 11.2 (22)
	(8–25)	(6–29)	(13–43)	(18–34)	(56–95)
**Overall**	12.4 ± 4.4 (59)	13.9 ± 6.8 (67)	26.7 ± 9.7 (47)	31.5 ± 10.0 (20)	75.6 ± 10.7 (45)
	(5–25)	(4–29)	(12–44)	(17–52)	(56–98)

## Discussion

Although firefly larvae can be detected by their glowing behavior, their life cycle and biology remain difficult to observe in natural habitats. *Ex-situ* observations are therefore important for understanding the biology of *Pteroptyx* fireflies, as larval development occurs within mangrove riverbank sediments. Basic aspects of the life cycle can sometimes be examined through short-term observations by collecting individuals at different developmental stages from the field and maintaining them briefly under controlled conditions, as reported for *Pteroptyx maipo* (Ballantyne et al., 2011). However, understanding the complete life cycle requires *in situ* breeding studies. Unfortunately, reliable rearing techniques for *Pteroptyx* fireflies have not yet been successfully developed for most species. To date, successful laboratory rearing has only been reported for two species, *Pteroptyx valida* (Jaikla, Thancharoen & Pinkaew, 2020; Ohba & Sim, 1994) and *Pteroptyx malaccae* (Loomboot et al., 2007). Foo, Sathiya Seelan & Dawood (2017) attempted to rear *Pteroptyx bearni* under laboratory conditions; however, the eggs became infected with microfungi, resulting in complete mortality and preventing hatching into first-instar larvae. These findings further indicate that the development of reliable laboratory rearing techniques for *Pteroptyx* fireflies remains challenging.

Most *ex-situ* studies of *Pteroptyx* have maintained fireflies under natural conditions, using substrates such as mangrove soil for oviposition and larval development (Foo, Sathiya Seelan & Dawood, 2017; Loomboot et al., 2007; Ohba & Sim, 1994), mosses as oviposition substrates (Nada & Kirton, 2004), and mangrove snails as larval food (Boonloi et al., 2025; Loomboot et al., 2007). These conditions were expected to allow the firefly stages to adapt and survive under controlled environments. However, some rearing techniques have also used artificial materials, such as sodium polyacrylate (Jaikla, Thancharoen & Pinkaew, 2020), wet tissue paper on activated carbon (Ballantyne et al., 2011), and moist cotton (Boonloi et al., 2025), which have successfully supported the life cycle of *Pteroptyx*. These materials can be advantageous for life-cycle studies because they provide a contrasting background against the firefly’s coloration, making observation and data recording easier. In this study, moist cotton was selected as the substrate for eggs and larvae and was replaced daily. Nevertheless, this material showed limitations during pupation. This consequently resulted in a low number of pupae and adults in this experiment. Most final-instar larvae did not appear to prefer cotton as a pupation substrate, and many pupae failed to emerge successfully as normal adults. Some pupae became entangled in the fine fibers of the cotton, resulting in the emergence of adults with abnormal wings. Under natural conditions, *Pteroptyx* prepupae construct a soil chamber within which pupation and the shedding of the larval cuticle occur. According to Weston & Desurmont (2008), key factors influencing pupation substrates include texture, moisture, and temperature, all of which contribute to maintaining a suitable microenvironment. Substrates that support the construction of a pupal chamber, such as soil, are therefore more suitable than those lacking this capacity, such as vermiculite and paper napkins (Barbosa-Andrade et al., 2017), consistent with the low pupation rate observed on moist cotton in this study. The pupal chamber may also play an important role in facilitating proper removal of the larval cuticle during metamorphosis, thereby supporting successful adult emergence. Similarly, elevated mortality during the prepupal to pupal transition has been reported in tiger beetles when individuals were transferred to artificial substrates (Cha, Jung & Knisley, 2025).

The complete life cycle of *Pteroptyx tener* was described in detail in this study, including the size and duration of each developmental stage, particularly all larval instars. Two trials were conducted to characterize the life cycle, and both exhibited broadly similar patterns in overall developmental duration, with the exception of L2 and L3. This variation may reflect differences in environmental or experimental conditions, particularly substrate moisture. These intermediate instars appear to be more sensitive to such conditions. However, they may exhibit developmental plasticity, allowing adjustment of developmental duration and thereby maintaining relatively high survival rates (88% and 76%, respectively) under both conditions. Variation in substrate moisture likely occurs naturally between wet and dry seasons, to which larvae must adapt to survive. In this study, the duration of L2 and L3 was shorter under higher moisture conditions (Trial 1) than under lower moisture conditions (Trial 2). These findings suggest that larval development of *Pteroptyx tener* may be prolonged under drier conditions in natural habitats, even when prey availability is high.

The life-cycle parameters observed here generally correspond with the preliminary findings of Nada & Kirton (2004), such as the duration of the egg stage, first-instar larva, pupa, and the overall life cycle. However, in contrast to their report, only five and six larval instars were observed in this study rather than seven. Variation in the number of larval instars may occur within a species due to environmental factors such as temperature, photoperiod, food quality, and humidity, as well as biological factors including sex and genetics (Esperk et al., 2007). Additional larval instars have also been reported in *Pteroptyx valida* (Jaikla, Thancharoen & Pinkaew, 2020) and in other firefly species, such as *Aquatica ficta* (Ho et al., 2010), with extra instars occurring more frequently in females.

A comparison between *Pteroptyx tener* in the present study and other *Pteroptyx* species indicates that the total life cycle of *Pteroptyx tener* is substantially longer than that reported for *Pteroptyx valida* (approximately 5 months) (Jaikla, Thancharoen & Pinkaew, 2020) and *Pteroptyx malaccae* (approximately 4 months) (Loomboot et al., 2007). However, such comparisons should be interpreted cautiously because developmental duration can be influenced by multiple factors, including species differences, temperature, humidity, diet, and other rearing conditions across studies. Nevertheless, the duration of the egg stage was comparable to that of *Pteroptyx valida*, and the pupal stage remained relatively short. Interestingly, the eggs of *Pteroptyx valida* were approximately 1.4 times larger than those of *Pteroptyx tener*.

The challenges of rearing *Pteroptyx tener* under controlled conditions are as follows: (1) the very small size of the eggs and first-instar larvae makes them difficult to handle and observe with the naked eye; (2) the pupation conditions remain unknown, although sterile mangrove soil or soil-like structure may be required; and (3) larvae are easily infected by nematodes, which can cause mortality. Based on the experience from this study, larvae collected from the field should be quarantined to prevent the spread of pathogens. Infection or stress during rearing may also cause abnormal development, resulting in larvae with wing-like structures (Fig. 2H).

Aggregation behavior in Coleopterans has been widely reported in many groups and may serve different ecological functions. For example, aggregation may function as an aposematic mechanism against predators in whirligig beetles (Vulinec & Miller, 1989), as predator dilution in bark beetles (Aukema & Raffa, 2004) or reflect environmental preferences in beetle species (Sun et al., 2023). Gregarious feeding behavior is also frequently observed in lepidopteran larvae (Qian et al., 2024; Reader & Hochuli, 2003). For instance, larvae of *Chlosyne janais* (Lepidoptera: Nymphalidae) exhibit gregarious behavior during early instars but become solitary feeders by the fourth instar (Denno & Benrey, 1997). Similarly, *Doratifera casta* larvae forage in large aggregations during early developmental stages, whereas mature larvae are typically solitary (Reader & Hochuli, 2003). Consistent with these patterns, first-instar larvae of *Pteroptyx tener* in the present study exhibited small-group feeding, particularly during the first week after hatching. This behavior may help young and vulnerable larvae maximize fitness by improving survival under conditions of low prey availability. Gregarious feeding may enhance larval survival and growth rates through cooperative or facilitative interactions among larvae (Qian et al., 2024). According to optimal foraging theory, predators maximize their fitness by balancing the energetic costs of predation with the nutritional value of prey in order to support rapid growth and maximize reproductive output (Jensen et al., 2012). Following Krämer, Hölker & Predel (2024), the firefly larvae inject midgut secretions for extra-oral digestion to paralyze snails. Gregarious feeding may reduce the energetic costs of prey capture and handling in first-instar larvae. However, intraspecific competition may also occur; therefore, large-group or mass aggregations were observed only under high larval densities and primarily during the first 2 weeks of the experiment, a pattern similar to that reported for *Necrodes littoralis* (Lis et al., 2024).

Generally, predatory insects require diets high in protein and relatively low in carbohydrates and lipids (Lalita & Gatoria, 2018). However, firefly larvae may also require substantial lipid reserves, as fat body tissues—particularly those associated with photogenic organs—have been documented in lampyrids (Tonolli et al., 2011). Because adult fireflies typically feed little or not at all, nutrient acquisition during the larval stage is critical for supporting metamorphosis, reproduction, and adult longevity. However, in this study, *Pteroptyx tener* larvae did not feed daily, likely because a single snail prey provides sufficient nutrients for several days and requires prolonged digestion, resulting in extended intervals between feeding events. Feeding cessation was also observed during pre-molt periods, typically lasting 1–3 days, although extended fasting of more than 27 days occasionally occurred. Such prolonged starvation may reduce pupation success (Jensen et al., 2012), and may therefore contribute to the low pupation rate observed in this study.

The feeding activity of *Pteroptyx tener* larvae suggests a generalist feeding habit, as they were able to utilize operculate juvenile golden apple snails, which are not part of their natural prey community, and consume them throughout all larval stages. The advantages of using juvenile freshwater snails as prey likely include their relatively thin shells, low mobility leading to shorter handling time, and relatively high protein content. However, larger larvae required an increasing number of snails to meet their nutritional demands, with a maximum of six snails consumed per larva per day in this study in third instar larvae (Fig. 6). Despite being readily accepted as prey, these snails may not represent an optimal diet for firefly development, as indicated by the relatively low pupation rate and reduced adult lifespan observed. In natural habitats, *Pteroptyx tener* larvae likely prey on a variety of snail species, which may allow them to balance the intake of key nutrients such as proteins and lipids to reach optimal levels for growth and reproduction, as predicted by nutritional foraging theory (Jensen et al., 2012), rather than relying on a single prey type. However, the nutritional requirements of *Pteroptyx tener* larvae have not been specifically identified.

### Implications for conservation of a vulnerable species

*Pteroptyx tener*, commonly known as the perfect synchronous flashing firefly, is currently classified as Vulnerable (VU B2ab (iii,v)) on the IUCN Red List, with a highly restricted area of occupancy (as low as 132 km^2^) and occurring in only 8–9 locations, with ongoing decline in habitat quality and population size (Nada et al., 2024b). In Thailand, *Pteroptyx tener* has been recorded from only a single locality, the Tapi River in Surat Thani Province (Sriboonlert et al., 2015), where it coexists with other *Pteroptyx* species, and its population has declined dramatically due to habitat alteration. According to Hobbs, Higgs & Harris (2009), the conservation and restoration of species are increasingly challenging due to rapid environmental changes driven by human activities, including land-use change, climate change, and species invasions, which have led to the emergence of novel ecosystems categorized as historical, hybrid, or novel depending on the extent of biotic and abiotic alteration. Likewise, the conservation and habitat restoration of *Pteroptyx tener* should adopt more flexible and adaptive strategies that consider how the species can coexist with human activities, while emphasizing ecosystem function, resilience, and the provision of ecosystem services.

The present study revealed several adaptive traits in *Pteroptyx tener*. First, eggs can be laid on artificial substrates, and larvae are able to survive under such conditions. Second, L1 exhibited gregarious feeding behavior, which may enhance survival during this weak and fragile stage. Third, developmental plasticity observed in intermediate instars (L2–L3) may allow larvae to adjust their developmental duration under changing environmental conditions. Fourth, *Pteroptyx tener* larvae appear to be generalist predators, capable of feeding on non-native operculate aquatic snails, indicating this species is not depending on specific snail species. Overall, these findings suggest that *Pteroptyx tener* possesses a degree of ecological flexibility, including the ability to develop on artificial substrates, exhibit gregarious feeding during the first instar, and utilize multiple snail species as prey. However, survival rates declined markedly in later instars (L4 and L5; 33% and 25%, respectively), including during the prepupal stage. These later instars are likely more sensitive to environmental conditions and may represent a critical bottleneck for the species.

Based on *in situ* observations, several key challenges to the conservation of *Pteroptyx tener* were identified. First, the minute size of early-instar larvae suggests that this stage may be particularly sensitive to environmental conditions and may represent a vulnerable phase in the life cycle. Second, larval development depends on a sufficient abundance of mangrove snails of varying sizes to support all larval stages. Although larvae can withstand temporary starvation, adequate prey availability is essential for proper growth and development. Third, nematode infection represents a potential biological threat, particularly affecting late-instar larvae. Attempts at *in situ* culture require intensive management yet yield low success rates. Therefore, habitat conservation and restoration are likely to represent the most effective strategies for the long-term conservation of *Pteroptyx tener*.

## Conclusions

This study provides a comprehensive account of the life cycle, morphology, and feeding ecology of *Pteroptyx tener* under laboratory conditions. The species exhibits a life cycle of approximately 7–8 months, with detailed characterization of the duration, morphology, and feeding ecology across all developmental stages. Survival remained high during early larval stages but declined markedly in later instars and during the prepupal transition, likely due to unsuitable pupation substrates. Gregarious feeding behavior was primarily observed in larvae less than 10 days old and was influenced by larval density. Pre-molt feeding cessation typically lasted 1–3 days, and total prey consumption reached approximately 76 snails per individual.

## Supplemental Information

10.7717/peerj.21575/supp-1 Supplemental Information 1 Body and protergum sizes of each larval instar.

10.7717/peerj.21575/supp-2 Supplemental Information 2 The habitat of *Pteroptyx tener* along Trang river, Thailand.

10.7717/peerj.21575/supp-3 Supplemental Information 3 Snail preparation for feeding firefly larvae.

## Additional Information and Declarations

### Field Study Permissions

The following information was supplied relating to field study approvals (*i.e*., approving body and any reference numbers):

The research was approved for animal care and use for scientific research at Kasetsart University (ACKU67-AGR-022), and the corresponding author obtained an animal use license (U1022522558).

### Data Availability

The following information was supplied regarding data availability:

The data is available in the Supplemental Files.
